# Human monocytes and macrophages regulate immune tolerance via integrin αvβ8–mediated TGFβ activation

**DOI:** 10.1084/jem.20171491

**Published:** 2018-11-05

**Authors:** Aoife Kelly, Sezin Gunaltay, Craig P. McEntee, Elinor E. Shuttleworth, Catherine Smedley, Stephanie A. Houston, Thomas M. Fenton, Scott Levison, Elizabeth R. Mann, Mark A. Travis

**Affiliations:** 1 Lydia Becker Institute of Immunology and Inflammation, Faculty of Biology, Medicine and Health, Manchester Academic Health Science Centre, University of Manchester, Manchester, UK; 2 Manchester Collaborative Centre for Inflammation Research, Faculty of Biology, Medicine and Health, Manchester Academic Health Science Centre, University of Manchester, Manchester, UK; 3 Wellcome Centre for Cell-Matrix Research, Faculty of Biology, Medicine and Health, Manchester Academic Health Science Centre, University of Manchester, Manchester, UK; 4 Gastroenterology Unit, Manchester Royal Infirmary, Manchester University National Health Service Foundation Trust, Manchester, UK; 5 Centre for Immunobiology, Institute of Infection, Immunity and Inflammation, College of Medicine, Veterinary Medicine and Life Sciences, University of Glasgow, Glasgow, UK

## Abstract

Monocytes are crucial immune cells involved in regulation of inflammation either directly or via differentiation into macrophages in tissues. However, many aspects of how their function is controlled in health and disease are not understood. Here we show that human blood monocytes activate high levels of the cytokine TGFβ, a pathway that is not evident in mouse monocytes. Human CD14^+^, but not CD16^+^, monocytes activate TGFβ via expression of the integrin αvβ8 and matrix metalloproteinase 14, which dampens their production of TNFα in response to LPS. Additionally, when monocytes differentiate into macrophages, integrin expression and TGFβ-activating ability are maintained in anti-inflammatory macrophages but down-regulated in pro-inflammatory macrophages. In the healthy human intestine, integrin αvβ8 is highly expressed on mature tissue macrophages, with these cells and their integrin expression being significantly reduced in active inflammatory bowel disease. Thus, our data suggest that integrin αvβ8–mediated TGFβ activation plays a key role in regulation of monocyte inflammatory responses and intestinal macrophage homeostasis.

## Abstract (precis)

TGFβ is a crucial immune regulator and attractive therapeutic target but needs to be activated to function. Kelly et al. show that human monocytes and macrophages activate TGFβ via expression of an integrin, αvβ8, which dampens pro-inflammatory cytokine production and is disrupted in inflammatory bowel disease.

## Graphical Abstract

**Figure:**
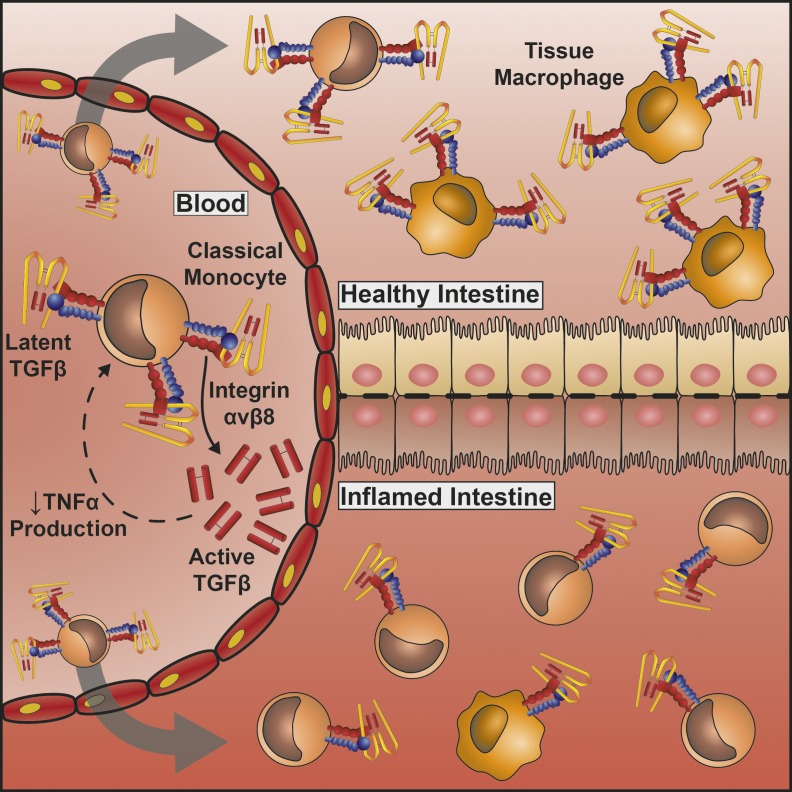

## Introduction

Monocytes are short-lived bone marrow–derived immune cells that play an important role in orchestrating inflammation but also promoting tolerance (Jakubzick et al., 2017). These cells are involved in phagocytosis, cytokine secretion, and antigen presentation but are probably best known for their role as a source of tissue macrophages. While not all macrophages are monocyte derived, with many being seeded at birth and maintained during life (Ginhoux and Guilliams, 2016), the intestine appears to be particularly reliant on peripheral monocytes as a source of intestinal macrophages (Bain et al., 2013, 2014). Although extensive work has been performed on pathways regulating murine monocyte and macrophage function, how these cells are regulated in humans and how such pathways are altered in inflammatory disease are less well understood.

TGFβ is a key multifunctional cytokine that is currently being targeted in a number of human diseases such as cancer and inflammatory disorders (Akhurst and Hata, 2012; Akhurst, 2017). An important function of TGFβ is its ability to regulate immunity, particularly T cell responses (Travis and Sheppard, 2014). While TGFβ is proposed to drive some effector T cell responses, specifically differentiation of Th9 (Dardalhon et al., 2008; Veldhoen et al., 2008) and Th17 cells (Korn et al., 2009), it is best known for its tolerogenic function (Kelly et al., 2017; Sanjabi et al., 2017). For example, TGFβ is important for promoting regulatory T cells (Chen et al., 2003; Davidson et al., 2007; Zheng et al., 2007; Liu et al., 2008) and blocking differentiation and function of Th1 (Gorham et al., 1998; Gorelik et al., 2002; Laouar et al., 2005) and Th2 cells (Gorelik et al., 2000; Heath et al., 2000; Kuwahara et al., 2012). Indeed, a key role for TGFβ in maintaining T cell homeostasis is shown by the observation that the TGFβ1 knockout mouse dies from CD4^+^ T cell–driven multi-organ inflammation early in life (Shull et al., 1992; Kulkarni et al., 1993). Although much is known about how TGFβ regulates T cell responses, much less is known about how TGFβ regulates innate immune responses, including regulation of monocytes and macrophages, and how such pathways are altered in disease.

TGFβ is expressed ubiquitously, but is always secreted as a latent complex, consisting of the latency-associated peptide (LAP) and active TGFβ moieties (Gleizes et al., 1997; Munger et al., 1997). Both LAP and active TGFβ are encoded by the *TGFB1* gene, are cleaved from each other intracellularly but remain noncovalently bound, and are secreted with LAP folded around active TGFβ to mask its TGFβ receptor binding sites (Munger et al., 1997). Thus, the function of TGFβ in the regulation of immune responses is controlled by mechanisms that regulate latent TGFβ activation. However, with many of the previous studies being performed in mice, how TGFβ is activated to control human immunity and how this is altered in disease settings are poorly understood.

Here we show that human monocytes and macrophages are key activators of latent TGFβ, with such pathways not present in mice. Mechanistically, this ability to activate TGFβ is dependent on expression of an integrin, αvβ8, with this activation important in dampening pro-inflammatory cytokine production by monocytes. Furthermore, we find that this pathway is highly expressed on human intestinal macrophages, which are reduced in inflammatory bowel disease (IBD) and replaced by incoming pro-inflammatory monocytes/macrophages that lack expression of αvβ8. Our data therefore highlight a new pathway by which human monocyte/macrophage function is regulated, which could be therapeutically targeted to modulate TGFβ-mediated control of innate immunity in inflammatory disease.

## Results and discussion

### Human CD14^+^ monocytes activate TGFβ via expression of the integrin αvβ8

**Figure 1.**
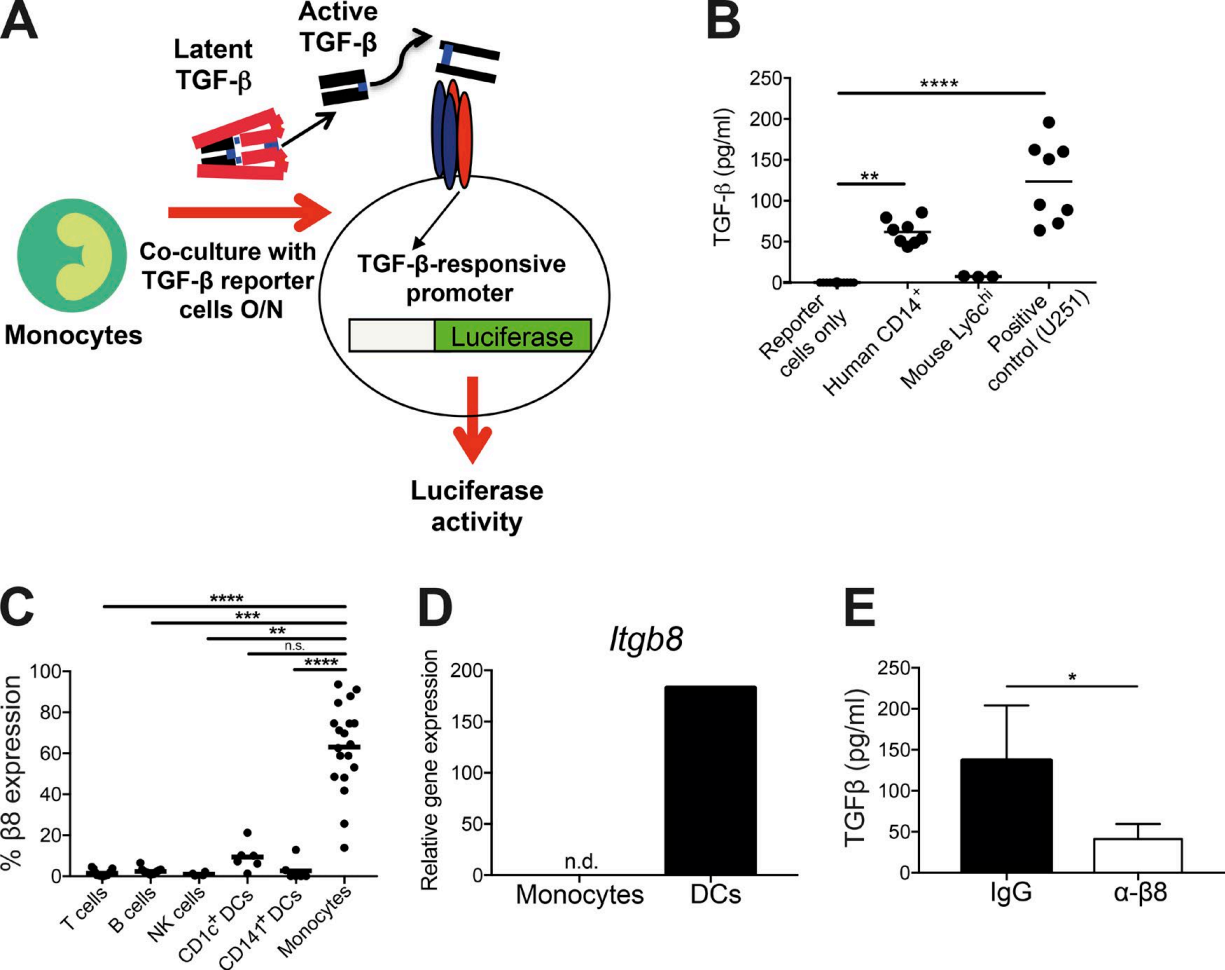
**Human peripheral blood monocytes activate TGFβ in an integrin αvβ8–dependent manner. (A)** Illustration of TGFβ activation assay whereby cells of interest are co-cultured with active TGFβ reporter cells (Abe et al., 1994) expressing a TGFβ-inducible promoter fused to luciferase. **(B)** Human CD14^+^ blood monocytes (*n* = 8) and murine splenic Ly6c^+^ monocytes (*n* = 3, each data point representing two pooled mice) were isolated and co-cultured with active TGFβ reporter cells overnight, before measuring luciferase activity (active TGFβ concentration calculated from standard curve). Reporter cells alone were used as a negative control (*n* = 8), and U251 cells (*n* = 8; Fenton et al., 2017) were used as a positive control. **(C)** Healthy human PBMCs were analyzed by flow cytometry. Following gating on single, live cells, integrin β8 expression was identified on lymphocyte populations (CD3 for T cells, CD19 for B cells, and CD56 for NK cells), DCs (lineage-negative [CD3, CD14, CD15, CD16, CD19, CD20, and CD56] HLA-DR^+^ CD11c^+^ with subsets identified by CD1c and CD141 staining), and monocytes (HLA-DR^+^, CD14^+^, and CD16^+^) following gating on forward and side scatter (*n* = 4–19). **(D)** RNA from murine spleen Ly6c^hi^ monocytes (lineage-negative [TCRβ, NK1.1, B220, CD19, Ter119, Ly6G, and Siglec F], CD11b^+^, Ly6c^+^) was analyzed for *Itgb8* expression by real-time PCR, normalizing to the housekeeping gene *Hprt* (*n* = 6, each from two pooled mice). Bone marrow–derived DCs were used as a positive control. **(E)** TGFβ activation of human CD14^+^ monocytes (*n* = 6) was measured in the presence of an isotype control antibody or an integrin β8 blocking antibody. Bars show mean and SD. n.s., not significant; n.d., not detected; *, P < 0.05; **, P < 0.01; ***, P < 0.001; ****, P < 0.0001; P values were determined by Kruskal–Wallis with Dunn’s multiple comparisons test (B and C) and Wilcoxon matched-pairs signed rank test (E).

Monocytes represent the major mononuclear phagocyte cell population in the periphery with a crucial role in mediating inflammatory responses via regulation of adaptive immunity and maturation into tissue macrophages. Given its major role in controlling immune responses, we hypothesized that TGFβ activation may be important for regulation of monocyte function. We therefore first assessed the ability of CD14^+^ monocytes from human blood to activate TGFβ via co-culture with an active TGFβ reporter cell line (Abe et al., 1994; Fig. 1 A). As a positive control for the assay, we used U251 cells, which we have previously shown to activate high levels of TGFβ via integrin αvβ8 (Fenton et al., 2017). We found that human CD14^+^ monocytes were potent activators of TGFβ (Fig. 1 B). Surprisingly, this ability was not present on murine monocytes, which did not activate any TGFβ above the baseline of reporter cells alone (Fig. 1 B), suggesting that this pathway is specific to humans. The failure to detect TGFβ activation by mouse monocytes was not due to an inability of the reporter cells to respond to murine TGFβ, as a near identical response was observed in the presence of recombinant active mouse and human TGFβ1 (Fig. S1 A). Additionally, the lack of TGFβ activation by mouse monocytes could not be explained by their inability to produce latent TGFβ, which was shown to be equivalent to human monocytes (Fig. S1 B).

Next, we looked to identify mechanisms responsible for the ability of human CD14^+^ monocytes to activate TGFβ. As we and others have recently shown that an integrin, αvβ8, is a key activator of TGFβ in the murine immune system (Lacy-Hulbert et al., 2007; Travis et al., 2007; Acharya et al., 2010; Païdassi et al., 2011; Worthington et al., 2011, 2013, 2015), we assessed expression of integrin αvβ8 on human peripheral immune cells by flow cytometry. As recently described (Fenton et al., 2017), integrin αvβ8 was expressed by CD1c^+^ but not CD141^+^ dendritic cells (DCs; Fig. 1 C). However, integrin αvβ8 was most highly expressed by blood monocytes, with ∼70% of these cells expressing the integrin (Fig. 1 C). In contrast, minimal expression of integrin αvβ8 was observed on lymphocyte populations (Fig. 1 C). In agreement with their lack of ability to activate TGFβ (Fig. 1 B), expression of integrin αvβ8 on murine Ly6c^hi^ monocytes isolated from spleen was undetectable, in contrast to expression on murine DCs (Fig. 1 D), which we have previously shown use integrin αvβ8 to activate TGFβ (Travis et al., 2007; Worthington et al., 2011).

To directly test whether expression of integrin αvβ8 is responsible for the ability of human blood monocytes to activate TGFβ, we used a recently described integrin β8 functional blocking antibody (Fenton et al., 2017). Treatment of monocytes with the integrin-blocking antibody almost completely ablated their ability to activate TGFβ (Fig. 1 E). These results show that human but not murine monocytes can activate high levels of the cytokine TGFβ, which is dependent on expression of the integrin αvβ8.

Previous results have shown that both mouse and human DCs can activate TGFβ via integrin αvβ8, suggesting some level of conservation in this pathway (Worthington et al., 2011; Fenton et al., 2017). However, our data now suggest that there is a new cellular pathway that regulates TGFβ activity in the human immune system, via monocyte expression of integrin αvβ8. Although our data show that murine monocytes are not capable of activating TGFβ, we cannot rule out that, in some biological contexts such as infection or inflammation, certain mouse monocyte subsets could activate TGFβ via integrin αvβ8–dependent or –independent pathways. However, the species-specific differences observed are in agreement with studies suggesting significant gene expression (Ingersoll et al., 2010) and functional (Gaidt et al., 2016) differences between human and mouse monocytes. Given the great interest in targeting TGFβ function in human diseases such as cancer and inflammation (Akhurst and Hata, 2012), it is crucial to understand human-specific pathways controlling the function of TGFβ.

### All human monocyte subsets express integrin αvβ8, but only CD14^+^ classical and intermediate monocytes activate TGFβ

In humans, distinct monocyte subsets can be identified based on expression of the surface proteins CD14 and CD16 (Ziegler-Heitbrock, 2015; Collin and Bigley, 2016). Thus, CD14^+^CD16^−^ classical monocytes represent 70–80% of blood monocytes, with CD14^+^CD16^+^ intermediate and CD14^−^CD16^+^ nonclassical monocyte subsets also existing, with recent data suggesting that human monocytes differentiate in a sequential manner from classical to intermediate and nonclassical monocytes (Patel et al., 2017). The different subsets are proposed to have distinct functions; for example, classical monocytes can produce pro-inflammatory cytokines and migrate to tissue sites where they differentiate into macrophages (Epelman et al., 2014; Ginhoux and Jung, 2014), whereas nonclassical CD16^+^ monocytes are thought to patrol the endothelium and be involved in anti-viral responses (Auffray et al., 2007; Cros et al., 2010). Therefore, we next determined whether there was heterogeneity in the ability of different human monocyte subsets to activate TGFβ.

**Figure 2.**
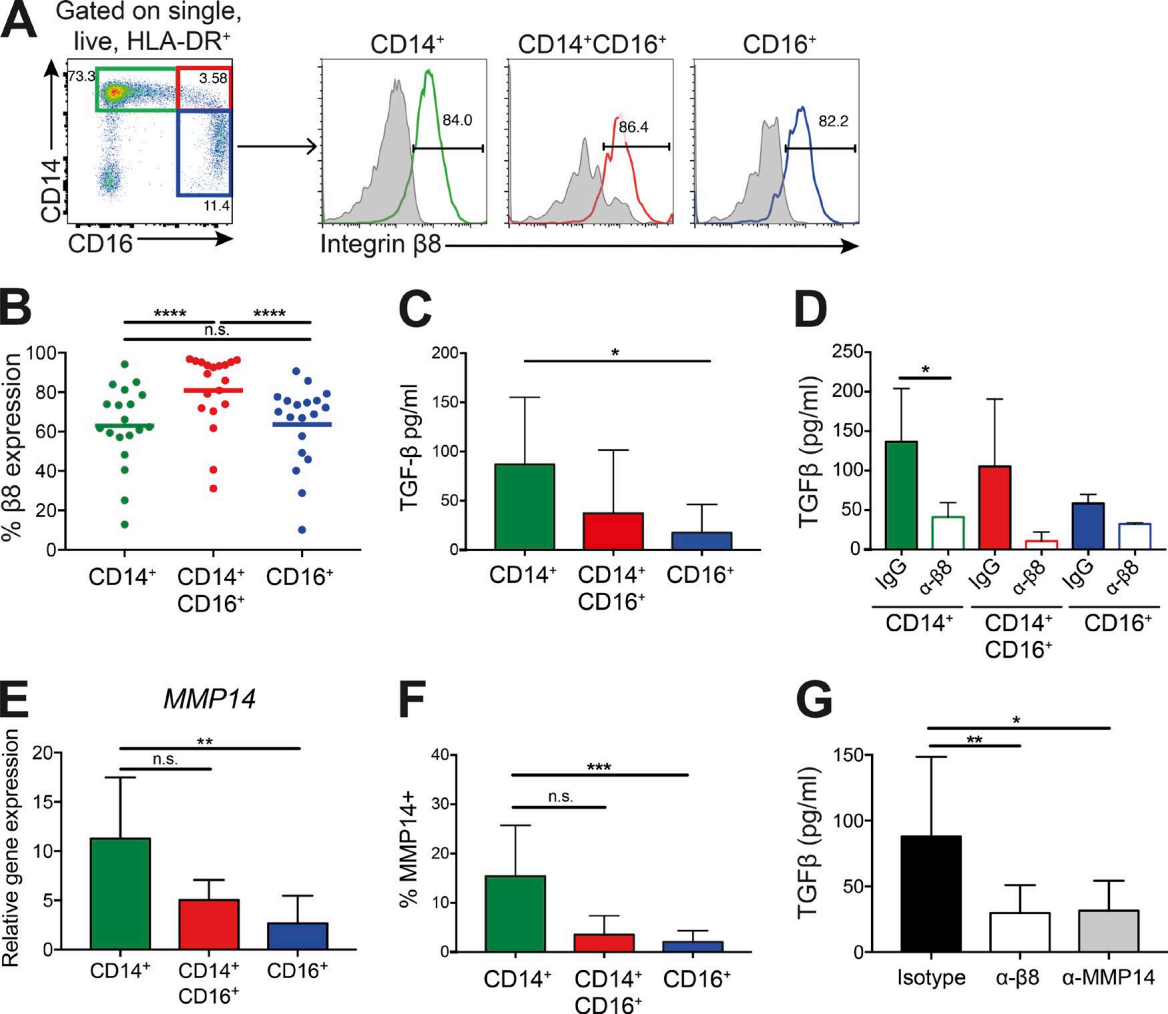
**The CD14^+^ classical monocyte subset activates high levels of TGFβ via integrin αvβ8 and MMP14. (A and B)** PBMCs were gated on single, live HLA-DR^+^ cells, then as CD14^+^CD16^−^ classical monocytes (green gate), CD14^+^CD16^+^ intermediate monocytes (red gate), and CD14^−^CD16^+^ nonclassical monocytes (blue gate). Levels of integrin β8 were measured on each subset (colored histogram, integrin β8 antibody; gray histogram, isotype control). Representative flow cytometry plots (A) and quantification of integrin β8 levels (B); *n* = 19 healthy donors. **(C and D)** Monocyte subsets were isolated by flow cytometry sorting and co-cultured overnight with a TGFβ reporter cell line (C; *n* = 10–13 donors) in the presence of an isotype or anti–integrin β8 antibody (D; *n* = 3–6 donors). Bars show mean and SD. **(E and F)** MMP14 expression was measured by real-time PCR, normalizing to the housekeeping gene *B2M* (E; *n* ≥ 5 for each subset) and flow cytometry (F; *n* = 8 for each subset) in monocyte subsets. Bars show mean and SD. **(G)** Active TGFβ production by CD14^+^ monocytes in the presence of an isotype control, integrin β8 blocking, or MMP14 blocking antibody (*n* = 6 donors). Bars show mean and SD. *, P < 0.05; **, P < 0.01; ***, P < 0.001; ****, P < 0.0001; n.s., not significant; P values were determined by Friedman test with Dunn’s multiple comparisons post-test (B, F, and G), Wilcoxon matched-pairs signed rank test (D), and Kruskal–Wallis with Dunn’s multiple comparisons post-test (C and E).

We first assessed integrin αvβ8 expression levels on different human monocyte subsets. Expression was similarly high on classical, intermediate, and nonclassical monocytes, with a slightly but significantly higher expression present on intermediate monocytes (Fig. 2, A and B), suggesting that all human monocyte subsets would be capable of activating TGFβ. However, although classical monocytes activated high levels of TGFβ, which was inhibited by an integrin αvβ8 functional blocking antibody, activation was almost completely absent by nonclassical monocytes, with intermediate levels of TGFβ activation observed by the intermediate monocyte subset (Fig. 2, C and D). Differences in TGFβ activation between monocytes could not be explained by differences in their ability to produce latent TGFβ, with all subsets expressing equivalent levels of latent TGFβ1 (Fig. S1 C). These data suggest that expression of integrin αvβ8 promotes TGFβ activation by classical monocytes, but that expression of the integrin is not sufficient to activate TGFβ in all monocyte subsets.

To determine mechanisms that promote differential TGFβ activation by monocyte subsets, despite all expressing high integrin αvβ8 levels, we focused on the expression of matrix metalloproteinase 14 (MMP14, also known as MT1-MMP), which has been suggested to synergize with integrin αvβ8 to promote TGFβ activation in some cells (Mu et al., 2002). Expression levels of MMP14 correlated with the ability of monocyte subsets to activate TGFβ via integrin αvβ8, with the highest levels seen in classical monocytes, and almost 10-fold lower levels in nonclassical monocytes (Fig. 2, E and F). To more directly assess the role for MMP14 in the ability of monocytes to activate TGFβ, we analyzed the ability of CD14^+^ monocytes to activate TGFβ in the presence or absence of an MMP14-blocking antibody. Inhibition of MMP14 resulted in a significant decrease in TGFβ activation, to similar levels observed in the presence of an integrin β8–blocking antibody (Fig. 2 G). Thus, overall, these data show an association between the expression of MMP14 and the ability of monocyte subsets to activate TGFβ, with classical monocytes requiring both integrin αvβ8 and MMP14 activity to promote TGFβ activation. Activation of TGFβ by other integrins, such as αvβ6, does not require MMP14 (Munger et al., 1999), and the ability of integrin αvβ8 to activate TGFβ in some cell types—for example, regulatory T cells (Stockis et al., 2017)—appears to be MMP14-independent. Here, our data suggest that expression of MMP14 at high levels on CD14^+^ monocytes likely cooperates with integrin αvβ8 to activate TGFβ via its enzymatic activity, a process that is lacking in other monocyte subsets.

### Integrin αvβ8–mediated TGFβ activation dampens pro-inflammatory cytokine production by human blood classical monocytes

**Figure 3.**
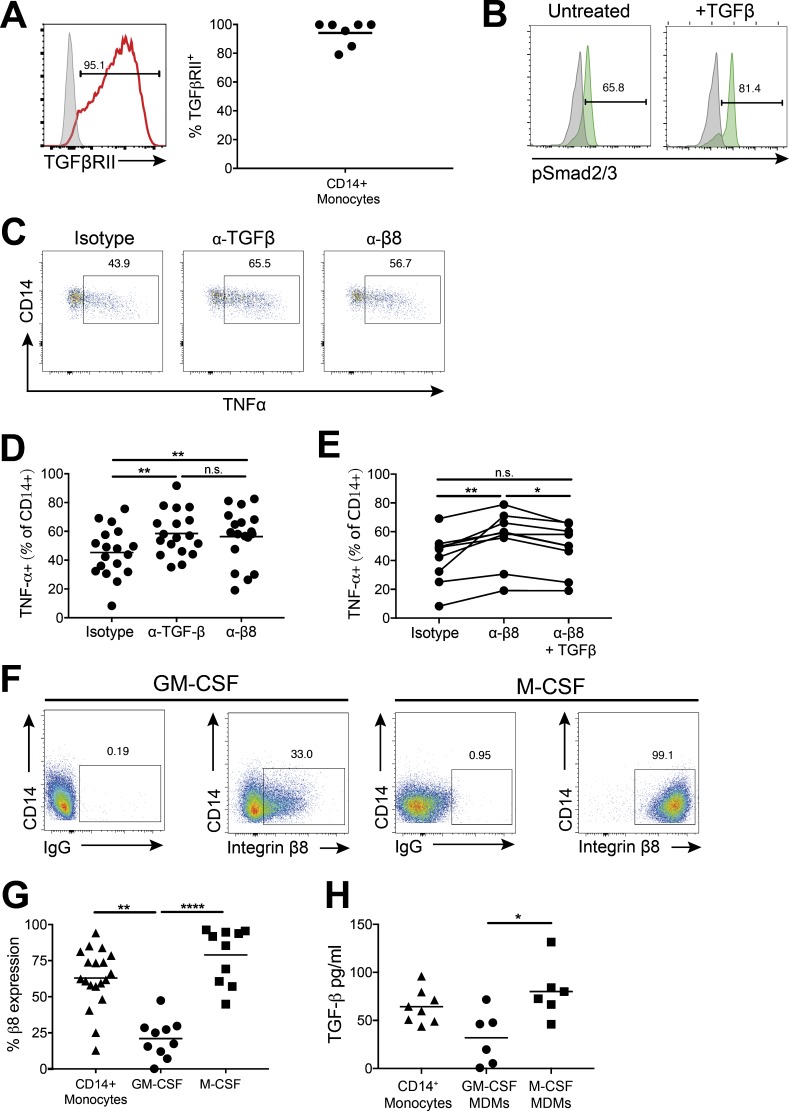
**Integrin αvβ8–mediated TGFβ activation reduces monocyte TNFα production in response to LPS and is down-regulated upon differentiation to pro-inflammatory macrophages. (A)** TGFβ receptor chain II (TGFβRII, red histogram) levels were assessed by flow cytometry on CD14^+^ monocytes (isotype control, gray histogram; graph shows cumulative data of *n* = 7 donors). **(B)** Levels of the TGFβ signaling molecule pSmad2/3 were measured in CD14^+^ classical monocytes (*n* = 3) after treatment of PBMCs with 5 ng/ml TGFβ for 30 min (isotype control shown in gray). Representative flow cytometry plots are displayed. **(C and D)** TNFα production by CD14^+^ monocytes was measured by intracellular flow cytometry, following PBMC culture overnight in the presence of an isotype control, anti-TGFβ, or anti–integrin β8 blocking antibody followed by activation with 10 ng/ml LPS for 4 h in the presence of a protein transport inhibitor; C shows representative flow cytometry plots; D shows cumulative data (*n* = 18). **(E)** TNFα production by CD14^+^ monocytes as in C and D, with addition of 5 ng/ml active TGFβ in the presence of anti–integrin β8 antibody (*n* = 9). **(F and G)** CD14^+^ monocytes were cultured for 6 d with GM-CSF or M-CSF to differentiate to macrophages and integrin β8 expression measured by flow cytometry; F displays representative flow cytometry plots for macrophage expression of integrin β8; G shows pooled data for monocyte and macrophage expression of integrin β8 from 10–19 donors. **(H)** Monocytes and MDMs were co-cultured overnight with active TGFβ reporter cells (*n* = 6–8 donors). *, P < 0.05; **, P < 0.01; ****, P < 0.0001, n.s., not significant; P values were determined by Friedman test with Dunn’s multiple comparisons post-test (D and E) or Kruskal–Wallis with Dunn’s multiple comparisons post-test (G and H).

We next investigated the functional importance of TGFβ activation by human monocytes, focusing on CD14^+^ classical monocytes, given that they activate the highest levels of TGFβ via integrin αvβ8 and MMP14. In support of a potential autocrine function for TGFβ activation by classical monocytes, these cells express high levels of the TGFβ receptor (Fig. 3 A), which is functionally active, as shown by phosphorylation of the TGFβ signaling protein Smad2/3 in response to active TGFβ (Fig. 3 B).

As monocytes are key, short-lived innate effector cells that can produce pro-inflammatory cytokines in response to pathogen challenge, and TGFβ can regulate cytokine production in other immune cells such as T cells (Kelly et al., 2017), we hypothesized that integrin αvβ8–mediated TGFβ activation may modulate the ability of monocytes to produce cytokines. To test this possibility, cells were cultured in the presence of blocking antibodies against TGFβ or integrin αvβ8 and then stimulated with LPS. In the presence of a TGFβ-blocking antibody, monocytes produced significantly increased levels of the pro-inflammatory cytokine TNFα (Fig. 3, C and D). Importantly, blockade of integrin αvβ8 yielded a similar increase in TNFα production by monocytes (Fig. 3, C and D), with this increase significantly reduced by addition of active TGFβ (Fig. 3 E).

Thus, these results indicate that integrin-mediated TGFβ activation normally functions to restrict CD14^+^ monocyte pro-inflammatory cytokine secretion. Such a function may act to potentiate over-activation of monocytes to prevent excessive inflammation. However, the functional importance of such a feedback loop requires further attention, especially in the light of recent data suggesting that, at least in the mouse, TNFα is an important survival factor for monocytes in steady state and inflammation (Wolf et al., 2017).

### Integrin αvβ8–mediated TGFβ activation is up-regulated on anti-inflammatory macrophages and down-regulated on pro-inflammatory macrophages

An important function of monocytes is the migration from the bone marrow and peripheral blood to tissue sites where they can differentiate into macrophages during homeostasis and inflammation (Ginhoux and Guilliams, 2016). We therefore tested whether the ability of monocytes to activate TGFβ was altered after differentiation into macrophages. To this end, we differentiated monocytes to macrophages in vitro, using the cytokines GM-CSF and M-CSF, which have been shown previously to give rise to pro-inflammatory and anti-inflammatory phenotypes, respectively (Murray et al., 2014). Indeed, in agreement with previous findings (Akagawa, 2002; Akagawa et al., 2006), we showed that GM-CSF–derived macrophages produced lower levels of IL-10 and were less phagocytic than M-CSF–derived macrophages (Fig. S2, A and B).

We found that pro-inflammatory GM-CSF–differentiated macrophages down-regulated expression of integrin αvβ8 compared with CD14^+^ monocytes, whereas monocytes differentiated to anti-inflammatory macrophages in the presence of M-CSF expressed high levels of integrin αvβ8, displaying close to 100% expression (Fig. 3, F and G). Additional polarization of GM-CSF–treated cells with IFN-γ and M-CSF–treated cells with IL-4, shown to further promote pro- and anti-inflammatory phenotypes, respectively (Murray et al., 2014), showed similar changes in integrin αvβ8 expression levels to GM-CSF and M-CSF treatment alone (Fig. S2, C–E). Expression of MMP14 was uniformly higher on both GM-CSF– and M-CSF–differentiated macrophages versus monocytes (Fig. S2 F), suggesting that signals received during the differentiation caused up-regulation of MMP14 expression.

These results suggest that macrophage populations may have differential abilities to activate TGFβ. Indeed, M-CSF–differentiated macrophages activated similar levels of TGFβ to CD14^+^ monocytes, whereas GM-CSF–differentiated macrophages showed a reduced ability to activate TGFβ (Fig. 3 H). Thus, our data show that anti-inflammatory macrophages are able to activate TGFβ, whereas this ability is down-regulated on more pro-inflammatory macrophage populations. Indeed, these results fit with previous findings suggesting that TGFβ is an important “M2” anti-inflammatory macrophage marker (Italiani and Boraschi, 2014).

### Human intestinal macrophages express high levels of integrin αvβ8 and are reduced in patients with IBD

**Figure 4.**
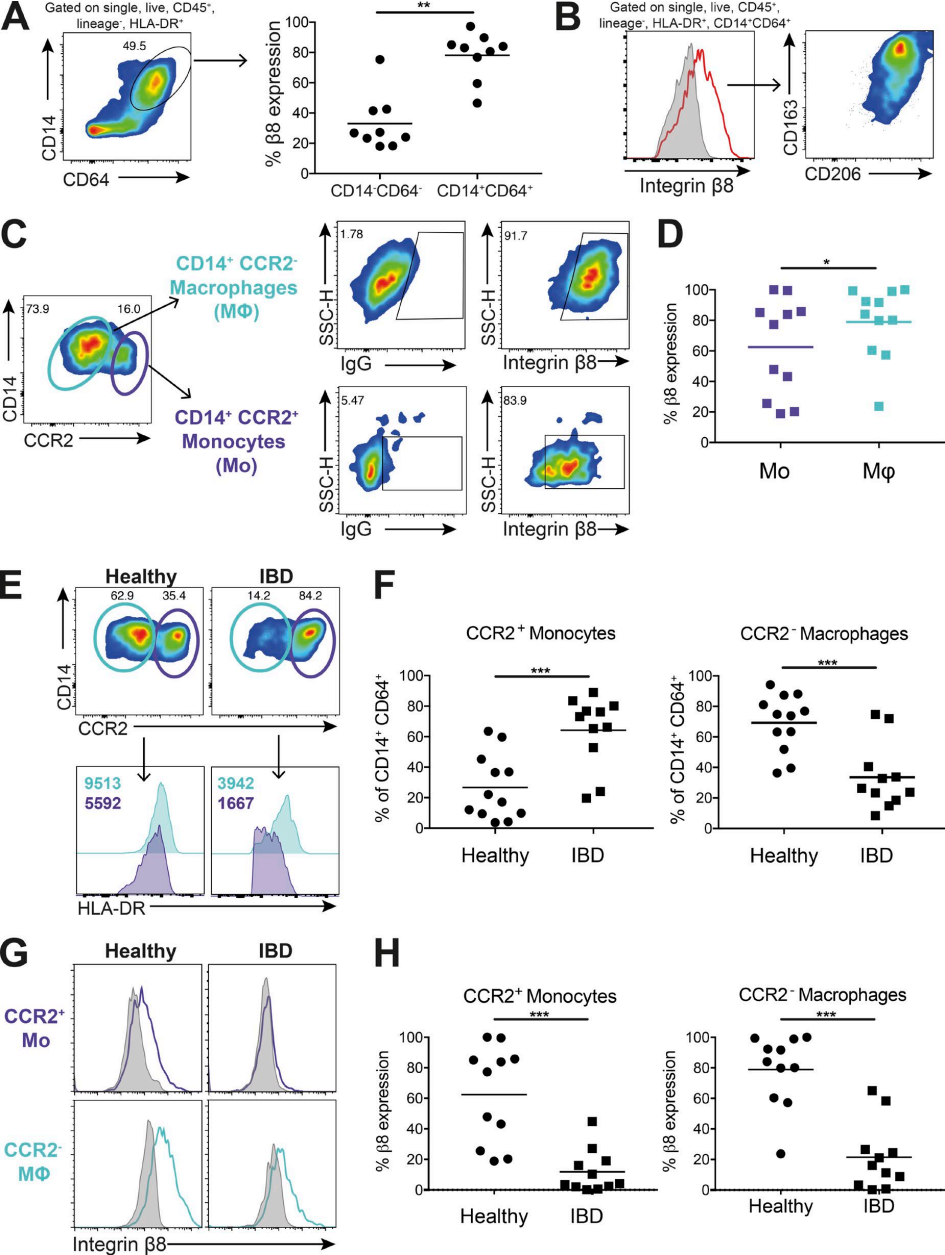
**Integrin αvβ8 is highly expressed on intestinal macrophages and is decreased in patients with IBD. (A)** Integrin β8 levels were analyzed by flow cytometry on human colonic monocyte/macrophage populations, gated as single, live, CD45^+^, lineage-negative (CD3, CD15, CD19, CD20, and CD56), HLA-DR^+^CD14^+^CD64^+^ cells. Expression of integrin αvβ8 was compared with equivalent CD14^−^CD64^−^ cells (*n* = 9). **(B)** Integrin β8^+^ monocytes/macrophages were analyzed for expression of the tissue macrophage markers CD163 and CD206 by flow cytometry (representative image from *n* = 9 donors). **(C and D)** Human monocyte/macrophage populations, identified as described in A, were gated for expression of the monocyte marker CCR2 and expression of integrin β8 on monocytes (CD14^+^CCR2^+^) and macrophages (CD14^+^CCR2^−^) analyzed. Representative flow cytometry plots (C) and cumulative data (D) from 11 donors are depicted. SSC-H, side scatter height. **(E)** Monocyte and macrophage populations in the intestinal tissue of healthy versus inflamed IBD patients were analyzed by flow cytometry (gated as described in C and D) with histograms showing levels of HLA-DR expression on monocytes (purple) and macrophages (blue) in healthy tissue compared with IBD. Representative plots shown, with F showing cumulative data for CCR2^+^ monocyte and CCR2^–^ macrophage levels from 11–12 healthy or IBD donors. **(G and H)** Expression levels of integrin αvβ8 were measured on monocyte/macrophage populations from healthy patients and patients with IBD. Representative histograms (G) and cumulative data (H) from 11 healthy and IBD donors are depicted. Bars show mean and SD. *, P < 0.05; **, P < 0.01; ***, P < 0.001; P values were determined by Wilcoxon matched-pairs signed rank test (A and D) or Mann–Whitney (F and H).

While in vitro–derived macrophages are a useful model, macrophages exist in multiple different phenotypes in vivo (Xue et al., 2014). In healthy tissue, anti-inflammatory macrophages capable of maintaining homeostasis are common, whereas a switch to pro-inflammatory monocytes/macrophages occurs during infection and inflammation (Varol et al., 2015). Given the proposed importance of tolerogenic macrophages in the intestine and the fact that intestinal macrophages derive predominantly from blood monocytes (Bain et al., 2014), coupled with data suggesting that TGFβ is a crucial cytokine in maintenance of immune homeostasis in the gut (Konkel and Chen, 2011; Kelly et al., 2017), we next assessed expression of integrin αvβ8 on human intestinal tissue macrophages. We found that intestinal macrophages, identified by gating on CD45^+^ lineage-negative, HLA-DR^+^CD14^+^CD64^+^ cells (Fig. S3 A), expressed high levels of integrin αvβ8 compared with CD64^−^ cells (Fig. 4 A). These integrin αvβ8^+^ intestinal macrophages also express CD163 and CD206 (Fig. 4 B), which have been proposed as markers of anti-inflammatory intestinal macrophages (Bain et al., 2013). Notably, murine intestinal macrophages, like murine monocytes, do not express integrin αvβ8 (Fig. S3 B), again highlighting an important species-specific difference in pathways that activate TGFβ.

We also gated cells on the basis of CCR2, with CCR2^+^ cells proposed to represent recently emigrated intestinal monocytes that are yet to differentiate to macrophages (Bain and Mowat, 2014). Such cells are a minor fraction of total intestinal HLA-DR^+^CD14^+^CD64^+^ cells in the healthy human intestine (Fig. 4 C) and display low forward scatter/side scatter levels compared with CCR2^–^ cells (Fig. S3 C), further supporting that such cells represent a monocyte/immature macrophage population. We found that both CD14^+^CCR2^+^ monocyte and CD14^+^CCR2^−^ macrophage populations in the intestine expressed high integrin αvβ8 levels, with significantly higher levels found on tissue macrophages (Fig. 4, C and D). Thus, together these data suggest that integrin αvβ8 is highly expressed on monocytes that enter the healthy intestine, and remains high as cells develop into macrophages.

Having established that integrin αvβ8 is conserved between human peripheral CD14^+^ monocytes and intestinal macrophages in the healthy intestine, we next addressed whether the pathway was altered in individuals with IBD. We observed a shift in the balance of monocytes and macrophages in the inflamed versus healthy intestine, with a significant increase in a CD14^hi^ HLA-DR^dim^ CCR2^+^ population (Fig. 4, E and F), proposed from previous work in mouse and humans to represent an inflammatory monocyte population (Kamada et al., 2008; Bain et al., 2013; Ogino et al., 2013; Thiesen et al., 2014; Magnusson et al., 2016). This influx of monocytes substantially reduces the proportion of tissue macrophages present (Fig. 4, E and F). Importantly, whereas both the monocyte population and tissue macrophages display clear expression of integrin αvβ8 in the healthy intestine, expression of the integrin is significantly down-regulated on inflammatory monocytes and remaining tissue macrophages in IBD (Fig. 4, G and H). MMP14 expression was also present in both intestinal monocytes and macrophages in healthy tissue, but was not altered in cells from patients with IBD (Fig. S3 D). When patients were stratified into those with Crohn’s disease or ulcerative colitis, significant reductions in monocyte and macrophage integrin β8 expression were observed in both disorders (Fig. S3, E and F). Thus, our results show that integrin αvβ8 is highly expressed on monocyte/macrophage populations in the steady-state intestine, but during inflammation, populations of integrin αvβ8–negative monocytes predominate.

Previous data have shown that, although levels of total latent TGFβ are increased in the intestine in IBD (Babyatsky et al., 1996), TGFβ signaling is in fact decreased (Monteleone et al., 2001). This apparently paradoxical finding was proposed to be due to enhanced expression of the TGFβ signaling inhibitor Smad7 in IBD, with targeting of Smad7 promoting enhanced TGFβ signaling (Monteleone et al., 2001). Our data now suggest that a reduced ability to activate TGFβ in the inflamed intestine, via down-regulation of integrin αvβ8–expressing monocytes/macrophages, may contribute to the reduced ability to trigger TGFβ signaling in the inflamed intestine. Together with our data (Fig. 3, C–E) showing that integrin αvβ8 expression on monocytes is required for the dampening of pro-inflammatory cytokine expression, our work suggests that down-regulation of this autocrine regulatory pathway in inflammatory monocyte/macrophage populations may promote inflammation. Additionally, as recent work in mouse models has suggested that TGFβ can promote intestinal macrophage differentiation in the steady state (Schridde et al., 2017), reduction of TGFβ activation pathways during inflammation may help explain the reduced proportions of intestinal macrophage populations observed in IBD patients.

To conclude, we have demonstrated that human but not murine monocytes and macrophages are specialized to activate TGFβ via expression of integrin αvβ8, which plays a regulatory role in dampening LPS-induced pro-inflammatory cytokine responses. This pathway is present on intestinal monocyte/macrophage populations during health, but is dysregulated during intestinal inflammation, suggesting that the pathway may represent a key immune regulatory mechanism in inflammation. Thus, promoting monocyte/macrophage ability to activate TGFβ via integrin αvβ8 may be a valid therapeutic strategy in inflammatory disorders such as IBD. To achieve this, it will be important in the future to determine signals that lead to the down-regulation of integrin αvβ8 during inflammation, which could then be targeted to promote the pathway. Targeting integrin αvβ8 (via small molecule inhibitors or blocking antibodies) is also attractive in the treatment in fibrosis (given the pro-fibrotic properties of TGFβ) and cancer (given the anti-inflammatory properties of TGFβ in the tumor microenvironment). However, our new data now highlight previously unappreciated cell types that will also be targeted by blocking integrin αvβ8 in humans, which will need to be considered in future therapeutic strategies.

## Materials and methods

### Samples and ethics

Human samples were obtained according to the principles expressed in the Declaration of Helsinki and under local ethical guidelines, and approved by the North West National Ethics Service (reference no. 15/NW/0007). Intestinal tissue samples were obtained via the Central Manchester University Hospitals National Health Service (NHS) Foundation Trust Biobank from patients undergoing surgery or endoscopy for chronically active Crohn’s disease or ulcerative colitis. The diagnosis of IBD was confirmed by established clinical, radiological, and histological criteria. Specimens were collected from healthy areas of colorectal cancer patients or patients undergoing endoscopy as noninflammatory controls. All patients provided written, informed consent for the collection of tissue samples and subsequent analysis. Healthy blood was obtained from donors recruited locally (according to the University of Manchester ethics and guidelines) or from leukocyte cones sourced from the blood bank (National Blood Service, Manchester, UK). Patient demographic and clinical information is listed in Table 1.

**Table 1.** Demographic and clinical data for patients who donated intestinal samples analyzed in the study

Sample type	Gender	Tissue	Age (yr)	Diagnosis	IBD medication
**Healthy (non-IBD) samples**					
Resections	Male	Colon	73	Sigmoid colon cancer	N/A
	Male	Colon	73	Concurrent bladder and rectal cancer	N/A
	Female	Colon	74	Sigmoid adenocarcinoma	N/A
	Male	Colon	75	Colorectal cancer with liver metastases	N/A
	Female	Colon	63	Caecal tumor and bladder tumor	N/A
	Female	Colon	73	Adenocarcinoma	N/A
	Male	Colon	64	Adenocarcinoma	N/A
	Male	Colon	64	Adenocarcinoma	N/A
	Female	Colon	51	Colon cancer with metastases	N/A
	Male	Colon	40	Rectal carcinoma with metastases	N/A
	Male	Rectum	67	Rectal carcinoma	N/A
	Female	Colon	57	Colon cancer with metastases	N/A
	Female	Colon	61	Colonic polyp	N/A
Biopsies	Male	Colon	46	Healthy (screen for cancer)	N/A
	Female	Colon	61	Healthy (screen for cancer)	N/A
	Male	Colon	79	Healthy (screen for cancer)	N/A
**IBD patients**					
Resections	Female	Colon	31	Active Crohn’s disease	Azathioprine
	Female	Colon	21	Active Crohn’s disease	Vedolizumab, prednisolone
	Male	Colon	25	Active Crohn’s disease	Infliximab, methotrexate
	Female	Colon	47	Active Crohn's disease	Prednisolone, simethicone
	Female	Colon	59	Active Crohn’s disease	Azathioprine, adalimumab, methotrexate
	Male	Colon	51	Active ulcerative colitis	Adalimumab
	Male	Colon	25	Active ulcerative colitis	Azathioprine, mesalazine
Biopsies	Male	Rectum	25	Active Crohn’s disease	Adalimumab, methotrexate
	Female	Colon	23	Active Crohn’s disease	Infliximab
	Male	Rectum	17	Active ulcerative colitis	Infliximab
	Male	Rectum	57	Active ulcerative colitis	Prednisone, omeprazole, mesalazine

N/A, not applicable.

### Peripheral blood mononuclear cells (PBMCs) and monocyte isolation

PBMCs were separated from healthy donor blood by layering over Ficoll Paque Plus (GE Healthcare) before centrifugation. Monocytes were isolated from PBMCs by magnetic bead separation using CD14 microbeads (Miltenyi Biotech), as per the manufacturer’s instructions. Monocyte purity was consistently >95%. Monocyte subsets were isolated by pre-enrichment using a Pan-Monocyte Isolation Kit or HLA-DR bead isolation kit (both from Miltenyi Biotech) followed by sorting by flow cytometry based on CD14 and CD16 expression.

### Monocyte-derived macrophages (MDMs)

MDMs were differentiated in RPMI plus l-glutamine (Sigma-Aldrich) with 10% FCS (Life Technologies, Thermo Fisher Scientific) and 1% penicillin/streptomycin (Sigma-Aldrich) in 12-well plates at 0.5 × 10^6^/ml for 6–7 d. Recombinant human GM-CSF or M-CSF (Peprotech) was added at a concentration of 50 ng/ml. Fresh media and cytokines were added on day 3.

### Murine monocyte isolation

Monocytes were isolated from the spleens of wild-type C57BL/6 mice. Briefly, spleens were pressed through a 40-µM cell strainer and washed with RPMI. Red cells were lysed using red cell lysis buffer (Sigma-Aldrich), and cells pre-enriched using a Monocyte Isolation Kit (BM; Miltenyi Biotech) as per the manufacturer’s instructions. Fc receptor block of enriched cells, using TruStain FcX (BioLegend), was performed, followed by staining and sorting of cells using the FACS Aria (BD Biosciences) via gating on lineage-negative (TCRβ, NK1.1, B220, CD19, Ter119, Ly6G, and Siglec F), CD11b^+^, Ly6c^+^ monocytes. 7-AAD (BioLegend) was used to discriminate dead cells (see Table S1 for antibody details).

### Isolation of lamina propria mononuclear cells (LPMCs)

LPMCs were isolated from tissue by first removing muscle and fat and then washing in HBSS, penicillin-streptomycin, G418 (Melford), and 1 mM dithiothreitol (Sigma-Aldrich). Tissue was then washed three times in HBSS, penicillin-streptomycin, G418, and 1 mM EDTA (Sigma-Aldrich) to remove epithelial cells. The tissue was then cut into small pieces and digested in Collagenase A (Roche) in 10% RPMI, penicillin-streptomycin, and G418 with 60 U/ml DNase I (Roche) for 1 h in a shaking incubator at 37°C. After this time, the tissue was filtered through a 40-µM filter, and cells were analyzed by flow cytometry.

### Flow cytometry surface staining

PBMCs, monocytes, MDMs, and LPMCs were stained in PBS with 1% FCS and 0.1% sodium azide (FACS buffer) by first preincubating in 2% mouse serum (Invitrogen), followed by staining for 20 min on ice (see Table S1 for details of all antibodies used). Cells were analyzed using a LSR Fortessa cytometer (BD Biosciences). Data were analyzed using Flowjo v10.1 (TreeStar).

### Phosphorylated (p)Smad2/3 intracellular staining

For pSmad2/3 staining, 100 µl of whole blood (100 µl/test) was either untreated or stimulated with TGFβ (Peprotech) for 30 min and then fixed by adding 1 ml of prewarmed 1X Lyse/Fix Buffer (BD Biosciences). Surface staining was performed for 60 min at room temperature using HLA-DR, CD14, and CD16, followed by washing and permeabilization using ice-cold Perm buffer III (BD Biosciences) for 30 min on ice. Following permeabilization, intracellular staining was performed using pSmad2/3 antibody (BD Biosciences) for 60 min at room temperature. Samples were washed in FACS buffer and acquired on the LSR Fortessa flow cytometer on the same day.

### Monocyte intracellular cytokine staining

Isolated PBMCs were cultured at 2 × 10^6^/ml in 10% RPMI in a 96-well flat-bottomed plate overnight in the presence of mouse IgG (40 µg/ml; Sigma-Aldrich) and isotype control antibody (40 µg/ml; clone MOPC-21; BioXCell), TGFβ blocking antibody (40 µg/ml; clone 1D11; BioXCell), or integrin β8 blocking antibody (20 µg/ml; clone ADWA16; Fenton et al., 2017). In some experiments, 5 ng/ml active TGFβ (Peprotech) was also added to cultures overnight. Cells were then stimulated with 10 ng/ml LPS (L4391; Sigma-Aldrich) for 4 h in the presence of GolgiPlug protein transport inhibitor (BD Biosciences). Following stimulation, cells were stained with lineage markers (CD3, CD15, CD19, CD20, and CD56), HLA-DR, CD14, and CD16, permeabilized with 0.5% saponin (Sigma-Aldrich), and then stained for intracellular TNFα. Cells were washed in saponin, resuspended in FACS buffer, and acquired on the LSR Fortessa on the same day.

### TGFβ activation assay

Transformed mink lung epithelial cells transfected with a plasmid containing firefly luciferase complementary DNA downstream of a TGFβ-sensitive promoter (a gift from D. Rifkin, New York University, New York, NY; Abe et al., 1994) were seeded in 96-well flat-bottomed plates in DMEM containing 10% FCS for at least 3 h. Monocytes or MDMs were added in the same medium in the presence of mouse IgG (40 µg/ml; Sigma-Aldrich) and isotype control antibody (40 µg/ml; clone MOPC-21; BioXCell), TGFβ blocking antibody (40 µg/ml; clone 1D11; BioXCell), integrin β8 blocking antibody (20 µg/ml; clone ADWA16) as described previously (Worthington et al., 2011; Fenton et al., 2017), or MMP14 blocking antibody (MAB3328; Merck Millipore). Cells were co-cultured for 16–20 h, and luciferase was then detected using the Luciferase Assay System (Promega) using an Infinite M200 Pro instrument (Tecan). A standard curve of active TGFβ (Peprotech) was generated and used to calculate levels of active TGFβ from luminescence intensity observed. TGFβ activity was determined as sample values with anti-TGFβ antibody background values subtracted. For comparison of mouse and human active TGFβ, recombinant proteins were purchased from BioLegend.

### Real-time PCR

RNA was isolated using RNeasy Mini or Micro kits (Qiagen). cDNA was synthesized using a High-Capacity RNA-to-cDNA kit (Applied Biosystems), and PCR was performed with Taqman Universal Mastermix II (Applied Biosystems/Thermo Fisher Scientific) on a QuantStudio 12K Flex instrument (Applied Biosystems). Primers used were as follows (all from Applied Biosystems): mouse *Itgb8* primers (Mm00623991_m1), mouse *Tgfb1* primers (Mm01178820_m1), human *MMP14* primers (Hs01037003_g1), and human *TGFB1* primers (Hs00998133_m1). Expression levels were calculated relative to the control genes *B2M* (Hs00984230_m1) for human and *Hprt* (Mm01545399_m1) for mouse.

### IL-10 ELISA

96-well Nunc Maxisorp plates (eBioscience) were coated with anti–IL-10 capture antibody overnight (eBioscience). Following a blocking step with ELISA/ELISPOT diluent (eBioscience), supernatants from day 7 MDM cultures were incubated in wells followed by addition of a biotinylated IL-10 detection antibody (eBioscience). Avidin-HRP (eBioscience) was added and 3,3′,5,5′-tetramethylbenzidine (TMB) solution was used to detect peroxidase activity, followed by TMB stop solution (BioLegend). Absorbance readings were measured using an Infinite M200 Pro plate reader (Tecan).

### Phagocytosis assay

On day 7 of culture, MDM supernatants were removed and replaced with fresh media. pHrodo Red *E. Coli* Bioparticles Conjugate for Phagocytosis (Life Technologies/Thermo Fisher Scientific) was added and incubated for 1 h, with pretreatment of cells with 5 µg/ml of Cytochalasin D (Sigma-Aldrich) for 30 min before addition of pHrodo particles used as a negative control. Cells were washed, removed by gentle scraping, fixed in 2% formaldehyde (Sigma-Aldrich), and analyzed by flow cytometry using an LSR II flow cytometer (BD Biosciences).

### Statistical analysis

Data were analyzed using GraphPad Prism version 7.0 for Mac. Statistical differences were tested as described in figure legends. Data are displayed as mean or mean and SD, as described in the figure legends.

### Online supplemental material

Fig. S1 shows active TGFβ reporter cell responses to mouse and human TGFβ1, and expression of TGFβ1 by human and mouse monocytes, and human monocyte subsets. Fig. S2 shows functional characterization of different MDM models and integrin αvβ8 and MMP14 expression on MDMs differentiated with various cytokines. Fig. S3 shows the gating strategy for monocyte/macrophage populations in human intestinal tissue, expression of integrin β8 on murine intestinal macrophages, MMP14 expression by monocytes and macrophages in health versus IBD, and expression of integrin β8 in Crohn’s disease and ulcerative colitis patients. Table S1 is a list of monoclonal antibodies used in the study.

## Supplementary Material

Supplemental Materials (PDF)

Table S1 (Excel file)

## References

[bib1] Abe M., Harpel J.G., Metz C.N., Nunes I., Loskutoff D.J., and Rifkin D.B. 1994 An assay for transforming growth factor-beta using cells transfected with a plasminogen activator inhibitor-1 promoter-luciferase construct. Anal. Biochem. 216:276–284. 10.1006/abio.1994.1042 8179182

[bib2] Acharya M., Mukhopadhyay S., Païdassi H., Jamil T., Chow C., Kissler S., Stuart L.M., Hynes R.O., and Lacy-Hulbert A. 2010 αv Integrin expression by DCs is required for Th17 cell differentiation and development of experimental autoimmune encephalomyelitis in mice. J. Clin. Invest. 120:4445–4452. 10.1172/JCI43796 21099114 PMC2993596

[bib3] Akagawa K.S. 2002 Functional heterogeneity of colony-stimulating factor-induced human monocyte-derived macrophages. Int. J. Hematol. 76:27–34. 10.1007/BF02982715 12138892

[bib4] Akagawa K.S., Komuro I., Kanazawa H., Yamazaki T., Mochida K., and Kishi F. 2006 Functional heterogeneity of colony-stimulating factor-induced human monocyte-derived macrophages. Respirology. 11(s uppl 1):S32–S36. 10.1111/j.1440-1843.2006.00805.x 16423268

[bib5] Akhurst R.J. 2017 Targeting TGF-β Signaling for Therapeutic Gain. Cold Spring Harb. Perspect. Biol. 9:a022301 10.1101/cshperspect.a022301 28246179 PMC5630004

[bib6] Akhurst R.J., and Hata A. 2012 Targeting the TGFβ signalling pathway in disease. Nat. Rev. Drug Discov. 11:790–811. 10.1038/nrd3810 23000686 PMC3520610

[bib7] Auffray C., Fogg D., Garfa M., Elain G., Join-Lambert O., Kayal S., Sarnacki S., Cumano A., Lauvau G., and Geissmann F. 2007 Monitoring of blood vessels and tissues by a population of monocytes with patrolling behavior. Science. 317:666–670. 10.1126/science.1142883 17673663

[bib8] Babyatsky M.W., Rossiter G., and Podolsky D.K. 1996 Expression of transforming growth factors alpha and beta in colonic mucosa in inflammatory bowel disease. Gastroenterology. 110:975–984. 10.1053/gast.1996.v110.pm8613031 8613031

[bib9] Bain C.C., and Mowat A.M. 2014 The monocyte-macrophage axis in the intestine. Cell. Immunol. 291:41–48. 10.1016/j.cellimm.2014.03.012 24726741 PMC4217150

[bib10] Bain C.C., Scott C.L., Uronen-Hansson H., Gudjonsson S., Jansson O., Grip O., Guilliams M., Malissen B., Agace W.W., and Mowat A.M. 2013 Resident and pro-inflammatory macrophages in the colon represent alternative context-dependent fates of the same Ly6Chi monocyte precursors. Mucosal Immunol. 6:498–510. 10.1038/mi.2012.89 22990622 PMC3629381

[bib11] Bain C.C., Bravo-Blas A., Scott C.L., Perdiguero E.G., Geissmann F., Henri S., Malissen B., Osborne L.C., Artis D., and Mowat A.M. 2014 Constant replenishment from circulating monocytes maintains the macrophage pool in the intestine of adult mice. Nat. Immunol. 15:929–937. 10.1038/ni.2967 25151491 PMC4169290

[bib12] Chen W., Jin W., Hardegen N., Lei K.J., Li L., Marinos N., McGrady G., and Wahl S.M. 2003 Conversion of peripheral CD4+CD25- naive T cells to CD4+CD25+ regulatory T cells by TGF-beta induction of transcription factor Foxp3. J. Exp. Med. 198:1875–1886. 10.1084/jem.20030152 14676299 PMC2194145

[bib13] Collin M., and Bigley V. 2016 Monocyte, Macrophage, and Dendritic Cell Development: the Human Perspective. Microbiol. Spectr. 4 10.1128/microbiolspec.MCHD-0015-2015 27780016

[bib14] Cros J., Cagnard N., Woollard K., Patey N., Zhang S.Y., Senechal B., Puel A., Biswas S.K., Moshous D., Picard C., 2010 Human CD14dim monocytes patrol and sense nucleic acids and viruses via TLR7 and TLR8 receptors. Immunity. 33:375–386. 10.1016/j.immuni.2010.08.012 20832340 PMC3063338

[bib15] Dardalhon V., Awasthi A., Kwon H., Galileos G., Gao W., Sobel R.A., Mitsdoerffer M., Strom T.B., Elyaman W., Ho I.C., 2008 IL-4 inhibits TGF-beta-induced Foxp3+ T cells and, together with TGF-beta, generates IL-9+ IL-10+ Foxp3(-) effector T cells. Nat. Immunol. 9:1347–1355. 10.1038/ni.1677 18997793 PMC2999006

[bib16] Davidson T.S., DiPaolo R.J., Andersson J., and Shevach E.M. 2007 Cutting Edge: IL-2 is essential for TGF-beta-mediated induction of Foxp3+ T regulatory cells. J. Immunol. 178:4022–4026. 10.4049/jimmunol.178.7.4022 17371955

[bib17] Epelman S., Lavine K.J., and Randolph G.J. 2014 Origin and functions of tissue macrophages. Immunity. 41:21–35. 10.1016/j.immuni.2014.06.013 25035951 PMC4470379

[bib18] Fenton T.M., Kelly A., Shuttleworth E.E., Smedley C., Atakilit A., Powrie F., Campbell S., Nishimura S.L., Sheppard D., Levison S., 2017 Inflammatory cues enhance TGFβ activation by distinct subsets of human intestinal dendritic cells via integrin αvβ8. Mucosal Immunol. 10:624–634. 10.1038/mi.2016.94 27782111 PMC5439516

[bib19] Gaidt M.M., Ebert T.S., Chauhan D., Schmidt T., Schmid-Burgk J.L., Rapino F., Robertson A.A., Cooper M.A., Graf T., and Hornung V. 2016 Human Monocytes Engage an Alternative Inflammasome Pathway. Immunity. 44:833–846. 10.1016/j.immuni.2016.01.012 27037191

[bib20] Ginhoux F., and Guilliams M. 2016 Tissue-Resident Macrophage Ontogeny and Homeostasis. Immunity. 44:439–449. 10.1016/j.immuni.2016.02.024 26982352

[bib21] Ginhoux F., and Jung S. 2014 Monocytes and macrophages: developmental pathways and tissue homeostasis. Nat. Rev. Immunol. 14:392–404. 10.1038/nri3671 24854589

[bib22] Gleizes P.E., Munger J.S., Nunes I., Harpel J.G., Mazzieri R., Noguera I., and Rifkin D.B. 1997 TGF-beta latency: biological significance and mechanisms of activation. Stem Cells. 15:190–197. 10.1002/stem.150190 9170210

[bib23] Gorelik L., Fields P.E., and Flavell R.A. 2000 Cutting edge: TGF-beta inhibits Th type 2 development through inhibition of GATA-3 expression. J. Immunol. 165:4773–4777. 10.4049/jimmunol.165.9.4773 11045997

[bib24] Gorelik L., Constant S., and Flavell R.A. 2002 Mechanism of transforming growth factor beta-induced inhibition of T helper type 1 differentiation. J. Exp. Med. 195:1499–1505. 10.1084/jem.20012076 12045248 PMC2193549

[bib25] Gorham J.D., Güler M.L., Fenoglio D., Gubler U., and Murphy K.M. 1998 Low dose TGF-beta attenuates IL-12 responsiveness in murine Th cells. J. Immunol. 161:1664–1670. 9712029

[bib26] Heath V.L., Murphy E.E., Crain C., Tomlinson M.G., and O’Garra A. 2000 TGF-beta1 down-regulates Th2 development and results in decreased IL-4-induced STAT6 activation and GATA-3 expression. Eur. J. Immunol. 30:2639–2649. 10.1002/1521-4141(200009)30:9<2639::AID-IMMU2639>3.0.CO;2-7 11009098

[bib27] Ingersoll M.A., Spanbroek R., Lottaz C., Gautier E.L., Frankenberger M., Hoffmann R., Lang R., Haniffa M., Collin M., Tacke F., 2010 Comparison of gene expression profiles between human and mouse monocyte subsets. Blood. 115:e10–e19. 10.1182/blood-2009-07-235028 19965649 PMC2810986

[bib28] Italiani P., and Boraschi D. 2014 From Monocytes to M1/M2 Macrophages: Phenotypical vs. Functional Differentiation. Front. Immunol. 5:514 10.3389/fimmu.2014.00514 25368618 PMC4201108

[bib29] Jakubzick C.V., Randolph G.J., and Henson P.M. 2017 Monocyte differentiation and antigen-presenting functions. Nat. Rev. Immunol. 17:349–362. 10.1038/nri.2017.28 28436425

[bib30] Kamada N., Hisamatsu T., Okamoto S., Chinen H., Kobayashi T., Sato T., Sakuraba A., Kitazume M.T., Sugita A., Koganei K., 2008 Unique CD14 intestinal macrophages contribute to the pathogenesis of Crohn disease via IL-23/IFN-gamma axis. J. Clin. Invest. 118:2269–2280. 18497880 10.1172/JCI34610 PMC2391067

[bib31] Kelly A., Houston S.A., Sherwood E., Casulli J., and Travis M.A. 2017 Regulation of Innate and Adaptive Immunity by TGFβ. Adv. Immunol. 134:137–233. 10.1016/bs.ai.2017.01.001 28413021

[bib32] Konkel J.E., and Chen W. 2011 Balancing acts: the role of TGF-β in the mucosal immune system. Trends Mol. Med. 17:668–676. 10.1016/j.molmed.2011.07.002 21890412 PMC3205325

[bib33] Korn T., Bettelli E., Oukka M., and Kuchroo V.K. 2009 IL-17 and Th17 Cells. Annu. Rev. Immunol. 27:485–517. 10.1146/annurev.immunol.021908.132710 19132915

[bib34] Kulkarni A.B., Huh C.G., Becker D., Geiser A., Lyght M., Flanders K.C., Roberts A.B., Sporn M.B., Ward J.M., and Karlsson S. 1993 Transforming growth factor beta 1 null mutation in mice causes excessive inflammatory response and early death. Proc. Natl. Acad. Sci. USA. 90:770–774. 10.1073/pnas.90.2.770 8421714 PMC45747

[bib35] Kuwahara M., Yamashita M., Shinoda K., Tofukuji S., Onodera A., Shinnakasu R., Motohashi S., Hosokawa H., Tumes D., Iwamura C., 2012 The transcription factor Sox4 is a downstream target of signaling by the cytokine TGF-β and suppresses T(H)2 differentiation. Nat. Immunol. 13:778–786. 10.1038/ni.2362 22751141 PMC3477402

[bib36] Lacy-Hulbert A., Smith A.M., Tissire H., Barry M., Crowley D., Bronson R.T., Roes J.T., Savill J.S., and Hynes R.O. 2007 Ulcerative colitis and autoimmunity induced by loss of myeloid alphav integrins. Proc. Natl. Acad. Sci. USA. 104:15823–15828. 10.1073/pnas.0707421104 17895374 PMC1994135

[bib37] Laouar Y., Sutterwala F.S., Gorelik L., and Flavell R.A. 2005 Transforming growth factor-beta controls T helper type 1 cell development through regulation of natural killer cell interferon-gamma. Nat. Immunol. 6:600–607. 10.1038/ni1197 15852008

[bib38] Liu Y., Zhang P., Li J., Kulkarni A.B., Perruche S., and Chen W. 2008 A critical function for TGF-beta signaling in the development of natural CD4+CD25+Foxp3+ regulatory T cells. Nat. Immunol. 9:632–640. 10.1038/ni.1607 18438410

[bib39] Magnusson M.K., Brynjólfsson S.F., Dige A., Uronen-Hansson H., Börjesson L.G., Bengtsson J.L., Gudjonsson S., Öhman L., Agnholt J., Sjövall H., 2016 Macrophage and dendritic cell subsets in IBD: ALDH+ cells are reduced in colon tissue of patients with ulcerative colitis regardless of inflammation. Mucosal Immunol. 9:171–182. 10.1038/mi.2015.48 26080709 PMC4683124

[bib40] Monteleone G., Kumberova A., Croft N.M., McKenzie C., Steer H.W., and MacDonald T.T. 2001 Blocking Smad7 restores TGF-beta1 signaling in chronic inflammatory bowel disease. J. Clin. Invest. 108:601–609. 10.1172/JCI12821 11518734 PMC209401

[bib41] Mu D., Cambier S., Fjellbirkeland L., Baron J.L., Munger J.S., Kawakatsu H., Sheppard D., Broaddus V.C., and Nishimura S.L. 2002 The integrin alpha(v)beta8 mediates epithelial homeostasis through MT1-MMP-dependent activation of TGF-beta1. J. Cell Biol. 157:493–507. 10.1083/jcb.200109100 11970960 PMC2173277

[bib42] Munger J.S., Harpel J.G., Gleizes P.E., Mazzieri R., Nunes I., and Rifkin D.B. 1997 Latent transforming growth factor-beta: structural features and mechanisms of activation. Kidney Int. 51:1376–1382. 10.1038/ki.1997.188 9150447

[bib43] Munger J.S., Huang X., Kawakatsu H., Griffiths M.J., Dalton S.L., Wu J., Pittet J.F., Kaminski N., Garat C., Matthay M.A., 1999 The integrin alpha v beta 6 binds and activates latent TGF beta 1: a mechanism for regulating pulmonary inflammation and fibrosis. Cell. 96:319–328. 10.1016/S0092-8674(00)80545-0 10025398

[bib44] Murray P.J., Allen J.E., Biswas S.K., Fisher E.A., Gilroy D.W., Goerdt S., Gordon S., Hamilton J.A., Ivashkiv L.B., Lawrence T., 2014 Macrophage activation and polarization: nomenclature and experimental guidelines. Immunity. 41:14–20. 10.1016/j.immuni.2014.06.008 25035950 PMC4123412

[bib45] Ogino T., Nishimura J., Barman S., Kayama H., Uematsu S., Okuzaki D., Osawa H., Haraguchi N., Uemura M., Hata T., 2013 Increased Th17-inducing activity of CD14+ CD163 low myeloid cells in intestinal lamina propria of patients with Crohn’s disease. Gastroenterology. 145:1380–91.e1. 10.1053/j.gastro.2013.08.049 23993972

[bib46] Païdassi H., Acharya M., Zhang A., Mukhopadhyay S., Kwon M., Chow C., Stuart L.M., Savill J., and Lacy-Hulbert A. 2011 Preferential expression of integrin αvβ8 promotes generation of regulatory T cells by mouse CD103+ dendritic cells. Gastroenterology. 141:1813–1820. 10.1053/j.gastro.2011.06.076 21745448 PMC3202651

[bib47] Patel A.A., Zhang Y., Fullerton J.N., Boelen L., Rongvaux A., Maini A.A., Bigley V., Flavell R.A., Gilroy D.W., Asquith B., 2017 The fate and lifespan of human monocyte subsets in steady state and systemic inflammation. J. Exp. Med. 214:1913–1923. 10.1084/jem.20170355 28606987 PMC5502436

[bib48] Sanjabi S., Oh S.A., and Li M.O. 2017 Regulation of the Immune Response by TGF-β: From Conception to Autoimmunity and Infection. Cold Spring Harb. Perspect. Biol. 9:a022236 10.1101/cshperspect.a022236 28108486 PMC5453394

[bib49] Schridde A., Bain C.C., Mayer J.U., Montgomery J., Pollet E., Denecke B., Milling S.W.F., Jenkins S.J., Dalod M., Henri S., 2017 Tissue-specific differentiation of colonic macrophages requires TGFβ receptor-mediated signaling. Mucosal Immunol. 10:1387–1399. 10.1038/mi.2016.142 28145440 PMC5417360

[bib50] Shull M.M., Ormsby I., Kier A.B., Pawlowski S., Diebold R.J., Yin M., Allen R., Sidman C., Proetzel G., Calvin D., 1992 Targeted disruption of the mouse transforming growth factor-beta 1 gene results in multifocal inflammatory disease. Nature. 359:693–699. 10.1038/359693a0 1436033 PMC3889166

[bib51] Stockis J., Liénart S., Colau D., Collignon A., Nishimura S.L., Sheppard D., Coulie P.G., and Lucas S. 2017 Blocking immunosuppression by human Tregs in vivo with antibodies targeting integrin αVβ8. Proc. Natl. Acad. Sci. USA. 114:E10161–E10168. 10.1073/pnas.1710680114 29109269 PMC5703296

[bib52] Thiesen S., Janciauskiene S., Uronen-Hansson H., Agace W., Högerkorp C.M., Spee P., Håkansson K., and Grip O. 2014 CD14(hi)HLA-DR(dim) macrophages, with a resemblance to classical blood monocytes, dominate inflamed mucosa in Crohn’s disease. J. Leukoc. Biol. 95:531–541. 10.1189/jlb.0113021 24212097

[bib53] Travis M.A., and Sheppard D. 2014 TGF-β activation and function in immunity. Annu. Rev. Immunol. 32:51–82. 10.1146/annurev-immunol-032713-120257 24313777 PMC4010192

[bib54] Travis M.A., Reizis B., Melton A.C., Masteller E., Tang Q., Proctor J.M., Wang Y., Bernstein X., Huang X., Reichardt L.F., 2007 Loss of integrin alpha(v)beta8 on dendritic cells causes autoimmunity and colitis in mice. Nature. 449:361–365. 10.1038/nature06110 17694047 PMC2670239

[bib55] Varol C., Mildner A., and Jung S. 2015 Macrophages: development and tissue specialization. Annu. Rev. Immunol. 33:643–675. 10.1146/annurev-immunol-032414-112220 25861979

[bib56] Veldhoen M., Uyttenhove C., van Snick J., Helmby H., Westendorf A., Buer J., Martin B., Wilhelm C., and Stockinger B. 2008 Transforming growth factor-beta ‘reprograms’ the differentiation of T helper 2 cells and promotes an interleukin 9-producing subset. Nat. Immunol. 9:1341–1346. 10.1038/ni.1659 18931678

[bib57] Wolf Y., Shemer A., Polonsky M., Gross M., Mildner A., Yona S., David E., Kim K.W., Goldmann T., Amit I., 2017 Autonomous TNF is critical for in vivo monocyte survival in steady state and inflammation. J. Exp. Med. 214:905–917. 10.1084/jem.20160499 28330904 PMC5379969

[bib58] Worthington J.J., Czajkowska B.I., Melton A.C., and Travis M.A. 2011 Intestinal dendritic cells specialize to activate transforming growth factor-β and induce Foxp3+ regulatory T cells via integrin αvβ8. Gastroenterology. 141:1802–1812. 10.1053/j.gastro.2011.06.057 21723222 PMC3507624

[bib59] Worthington J.J., Klementowicz J.E., Rahman S., Czajkowska B.I., Smedley C., Waldmann H., Sparwasser T., Grencis R.K., and Travis M.A. 2013 Loss of the TGFβ-activating integrin αvβ8 on dendritic cells protects mice from chronic intestinal parasitic infection via control of type 2 immunity. PLoS Pathog. 9:e1003675 10.1371/journal.ppat.1003675 24098124 PMC3789784

[bib60] Worthington J.J., Kelly A., Smedley C., Bauché D., Campbell S., Marie J.C., and Travis M.A. 2015 Integrin αvβ8-Mediated TGF-β Activation by Effector Regulatory T Cells Is Essential for Suppression of T-Cell-Mediated Inflammation. Immunity. 42:903–915. 10.1016/j.immuni.2015.04.012 25979421 PMC4448149

[bib61] Xue J., Schmidt S.V., Sander J., Draffehn A., Krebs W., Quester I., De Nardo D., Gohel T.D., Emde M., Schmidleithner L., 2014 Transcriptome-based network analysis reveals a spectrum model of human macrophage activation. Immunity. 40:274–288. 10.1016/j.immuni.2014.01.006 24530056 PMC3991396

[bib62] Zheng S.G., Wang J., Wang P., Gray J.D., and Horwitz D.A. 2007 IL-2 is essential for TGF-beta to convert naive CD4+CD25- cells to CD25+Foxp3+ regulatory T cells and for expansion of these cells. J. Immunol. 178:2018–2027. 10.4049/jimmunol.178.4.2018 17277105

[bib63] Ziegler-Heitbrock L. 2015 Blood Monocytes and Their Subsets: Established Features and Open Questions. Front. Immunol. 6:423 10.3389/fimmu.2015.00423 26347746 PMC4538304

