# Supplementary material for: Human monocytes and macrophages regulate immune tolerance via integrin αvβ8–mediated TGFβ activation
Source: J Exp Med. 2018 Nov 5;215(11):2725–36. doi: 10.1084/jem.20171491 (PMC6219736; doi:10.1084/jem.20171491)
Supplement: Supplemental Materials (PDF) [file JEM_20171491_sm.pdf]

Supplemental material

Kelly et al., https://doi.org/10.1084/jem.20171491

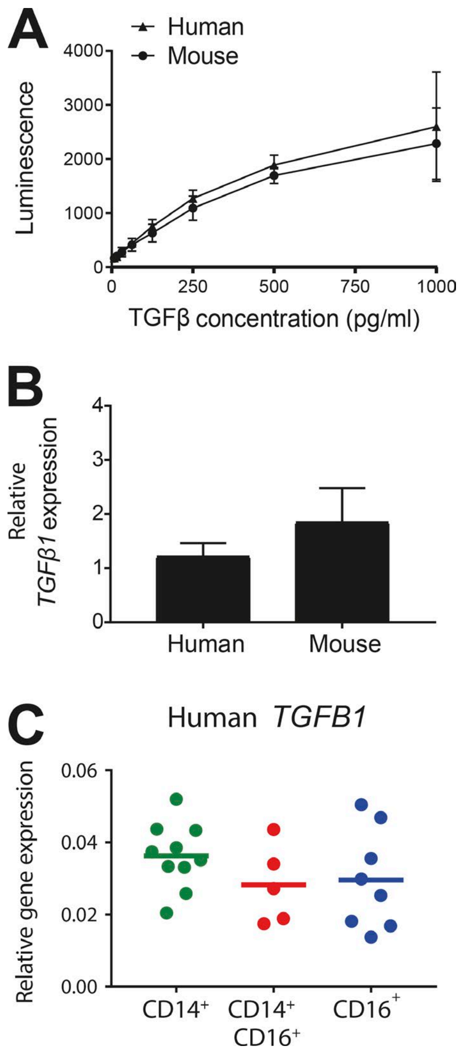

Figure S1. **Active TGFβ reporter cells respond equally to murine and human TGFβ1, and different monocyte subsets show equivalent TGFβ1 expression. (A)** Active TGFβ reporter cells (Abe et al., 1994) were cultured overnight in the presence of doses of murine or human active TGFβ1 before measurement of luciferase signal (n = 3). **(B)** Human $CD14^+$ monocytes were sorted from peripheral blood, and murine $Ly6C^{hi}$ monocytes were sorted from spleen. *TGFB1* expression was measured by real-time PCR in monocyte subsets, normalizing to the housekeeping gene *B2M* for human samples and *Hprt* for mouse samples. Data are representative of two independent experiments, $n \geq 6$. Data shown are means; error bars are SD. **(C)** Human monocyte subsets were sorted from peripheral blood and *TGFB1* expression measure by real-time PCR, normalizing to the housekeeping gene *B2M*. n = 5–10.

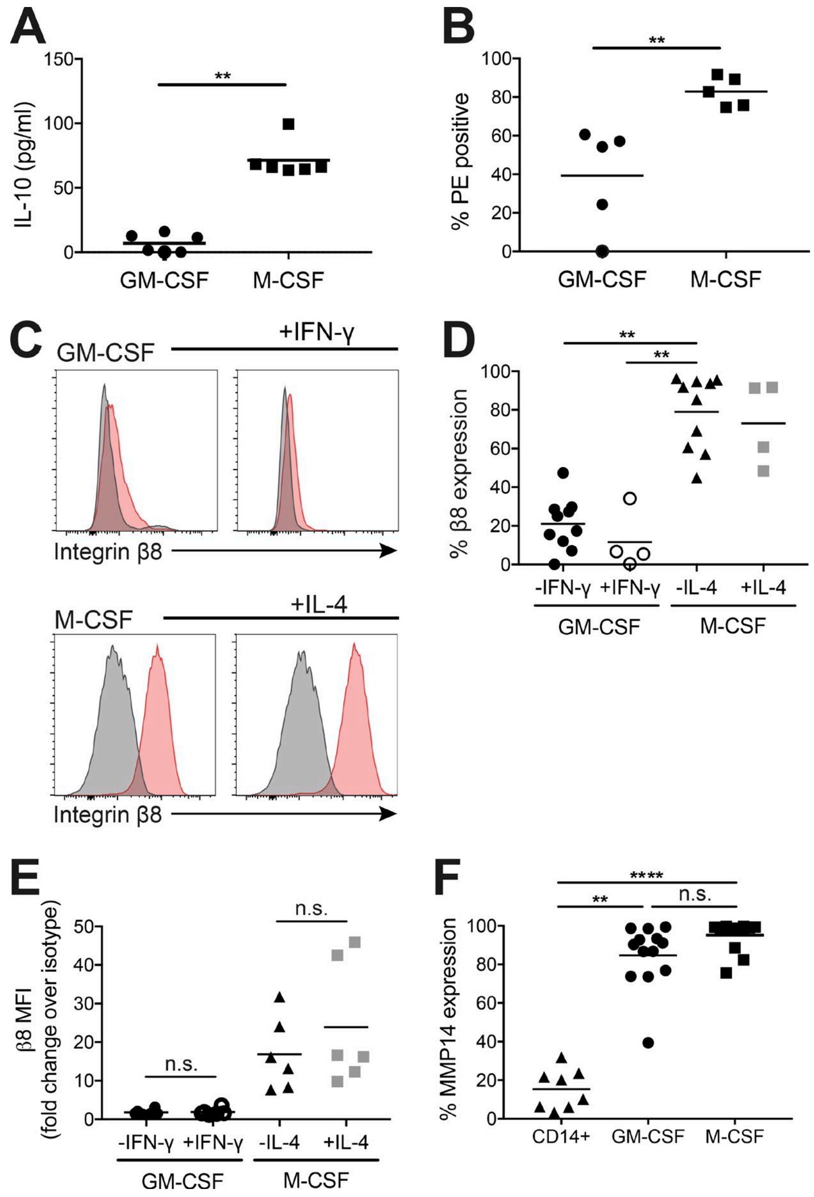

Figure S2. **Integrin β8 expression is highest on M-CSF MDMs showing increased IL-10 production and phagocytic ability. (A)** IL-10 production was measured in supernatants from GM-CSF– and M-CSF–derived MDMs cultured for 6 d (*n* = 6). **(B)** The capacity of GM-CSF– and M-CSF–derived MDMs to phagocytose PE-labeled *Escherichia coli* was compared (*n* = 5). **(C)** Representative histograms showing integrin β8 expression (red) on MDMs derived using GM-CSF$^{+/-}$ IFN-γ or M-CSF$^{+/-}$ IL-4. Isotype control is shown in gray. **(D and E)** Graph shows cumulative data from *n* = 4–10 donors (D, percent expression; E, mean fluorescence intensity [MFI]). **(F)** Expression of MMP14 in CD14$^+$ monocytes, GM-CSF–differentiated MDMs, and M-CSF–differentiated MDMs was analyzed by flow cytometry (*n* = 8–12). **, P < 0.01; ****, P < 0.001, n.s., not significant; P values were calculated using Mann–Whitney (A and B) and Kruskal–Wallis with Dunn's multiple comparison post-test (D–F).

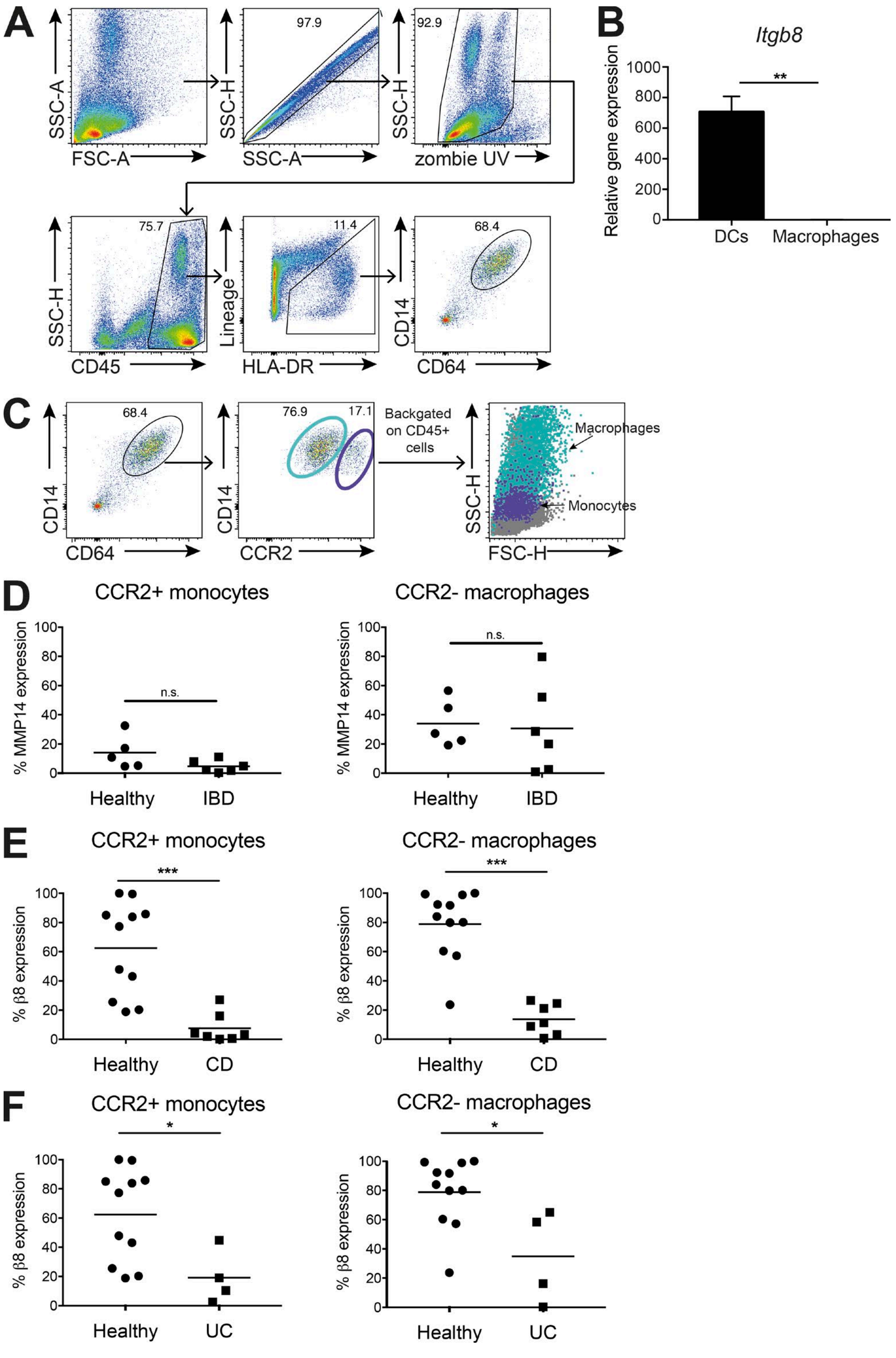

Figure S3. **Characterization of integrin αvβ8 and MMP14 expression by intestinal monocyte/macrophage populations in healthy versus inflamed IBD tissue. (A)** LPMCs were isolated from healthy intestinal tissue and analyzed by flow cytometry via gating on single, live, $CD45^+$, lineage-negative (CD3, CD15, CD19, CD20, and CD56), HLA-DR^+ cells within which monocyte/macrophage populations were identified by gating on $CD14^+CD64^+$ cells. **(B)** Expression of integrin β8 in murine intestinal macrophages. Macrophages were isolated from the intestine as live $CD45^+SiglecF^-Ly6G^-CD64^+CD11b^+Ly6C^-MHCII^+$ cells, and *Itgb8* mRNA levels were measured by real-time PCR. Intestinal DCs (live $CD45^+B220^-CD64^-MHCII^+CD11c^+CD103^+CD11b^-$) were used as a positive control. $n = 3$. **(C)** Intestinal monocyte/macrophage populations, gated as in A, were distinguished via CCR2 expression, and populations were backgated on $CD45^+$ cells to demonstrate the forward scatter (FSC) and side scatter (SSC) profile of monocytes (purple) and macrophages (blue). **(D)** Intestinal monocytes/macrophages, gated as described in A, then as $CCR2^+$ monocytes or $CCR2^-$ macrophages, were analyzed by flow cytometry for expression of MMP14 in healthy tissue compared with inflamed tissue from IBD patients ($n = 5$–6). **(E and F)** Integrin β8 expression was determined by flow cytometry in healthy intestinal tissue compared with inflamed tissue from (E) Crohn's disease (CD) or (F) ulcerative colitis (UC) patients ($n = 4$–11). *, $P < 0.05$; **, $P \leq 0.01$; ***, $P < 0.005$; n.s., not significant. Statistics were calculated using Mann–Whitney tests.

Table S1 is provided as an Excel file and provides details of antibodies and reagents used for flow cytometry.

References

Abe, M., J.G. Harpel, C.N. Metz, I. Nunes, D.J. Loskutoff, and D.B. Rifkin. 1994. An assay for transforming growth factor-beta using cells transfected with a plasminogen activator inhibitor-1 promoter-luciferase construct. *Anal. Biochem.* 216:276–284. https://doi.org/10.1006/abio.1994.1042
